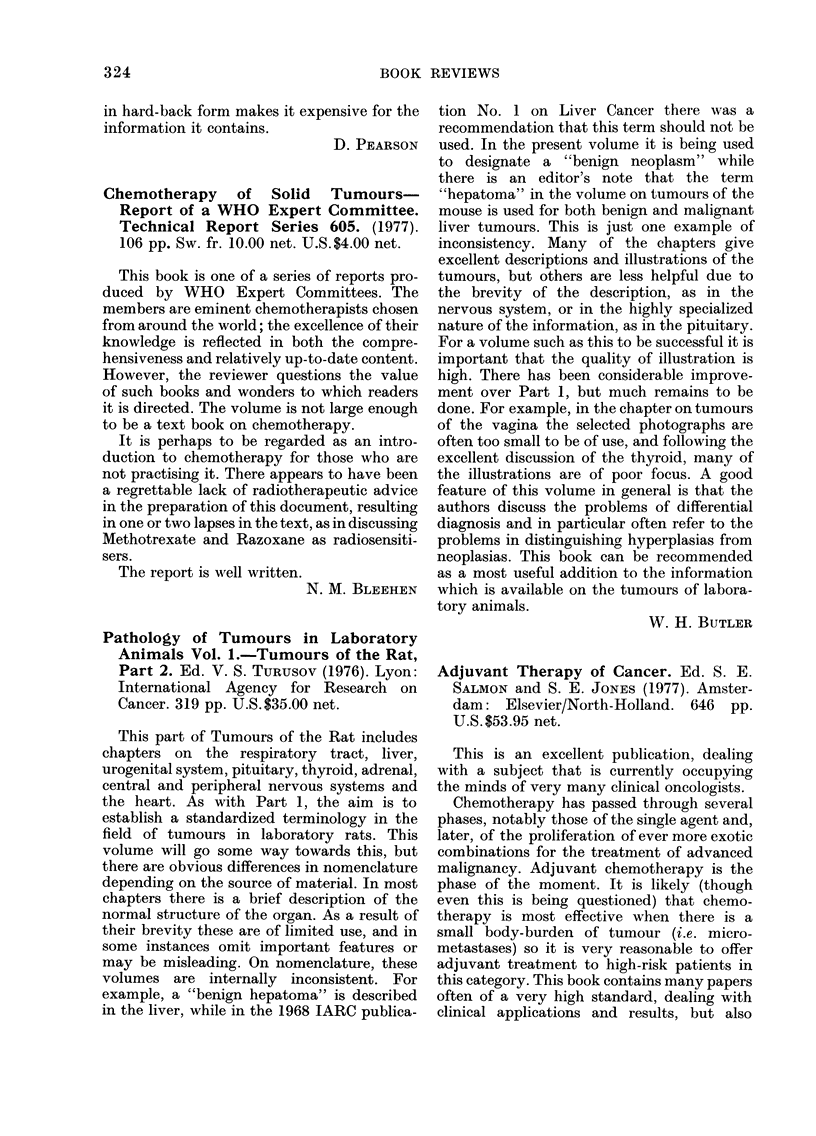# Pathology of Tumours in Laboratory Animals Vol. 1.—Tumours of the Rat, Part 2

**Published:** 1978-02

**Authors:** W. H. Butler


**Pathology of Tumours in Laboratory Animals Vol. 1.—Tumours of the Rat, Part 2.** Ed. V. S. Turusov (1976). Lyon: International Agency for Research on Cancer. 319 pp. U.S.$35.00 net.

This part of Tumours of the Rat includes chapters on the respiratory tract, liver, urogenital system, pituitary, thyroid, adrenal, central and peripheral nervous systems and the heart. As with Part 1, the aim is to establish a standardized terminology in the field of tumours in laboratory rats. This volume will go some way towards this, but there are obvious differences in nomenclature depending on the source of material. In most chapters there is a brief description of the normal structure of the organ. As a result of their brevity these are of limited use, and in some instances omit important features or may be misleading. On nomenclature, these volumes are internally inconsistent. For example, a "benign hepatoma" is described in the liver, while in the 1968 IARC publication No. 1 on Liver Cancer there was a recommendation that this term should not be used. In the present volume it is being used to designate a "benign neoplasm" while there is an editor's note that the term "hepatoma" in the volume on tumours of the mouse is used for both benign and malignant liver tumours. This is just one example of inconsistency. Many of the chapters give excellent descriptions and illustrations of the tumours, but others are less helpful due to the brevity of the description, as in the nervous system, or in the highly specialized nature of the information, as in the pituitary. For a volume such as this to be successful it is important that the quality of illustration is high. There has been considerable improvement over Part 1, but much remains to be done. For example, in the chapter on tumours of the vagina the selected photographs are often too small to be of use, and following the excellent discussion of the thyroid, many of the illustrations are of poor focus. A good feature of this volume in general is that the authors discuss the problems of differential diagnosis and in particular often refer to the problems in distinguishing hyperplasias from neoplasias. This book can be recommended as a most useful addition to the information which is available on the tumours of laboratory animals.

W. H. Butler